# Supplementary material for: The causal role of circulating inflammatory markers in osteoporosis: a bidirectional Mendelian randomized study
Source: Front Immunol. 2024 Jul 18;15:1412298. doi: 10.3389/fimmu.2024.1412298 (PMC11291241; doi:10.3389/fimmu.2024.1412298)
Supplement: Supplementary file 1 [file DataSheet_1.zip › supplementary material/CIM-OSTEOPOROSIS /GCST90274800.csv_leave_one_out.pdf]

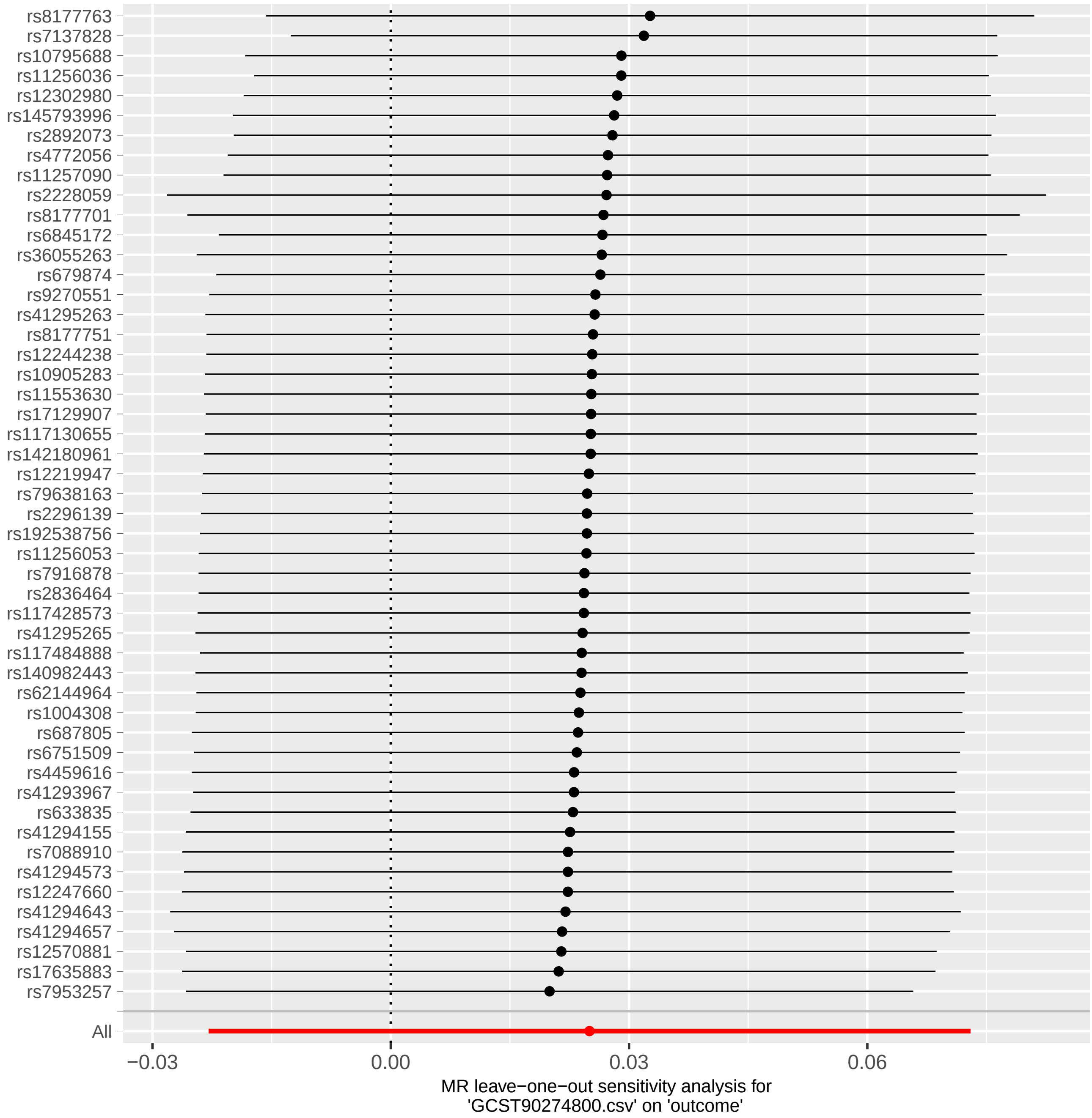
rs8177763
rs7137828
rs10795688
rs11256036
rs12302980
rs145793996
rs2892073
rs4772056
rs11257090
rs2228059
rs8177701
rs6845172
rs36055263
rs679874
rs9270551
rs41295263
rs8177751
rs12244238
rs10905283
rs11553630
rs17129907
rs117130655
rs142180961
rs12219947
rs79638163
rs2296139
rs192538756
rs11256053
rs7916878
rs2836464
rs117428573
rs41295265
rs117484888
rs140982443
rs62144964
rs1004308
rs687805
rs6751509
rs4459616
rs41293967
rs633835
rs41294155
rs7088910
rs41294573
rs12247660
rs41294643
rs41294657
rs12570881
rs17635883
rs7953257
All
−0.03
0.00
0.03
0.06
MR leave−one−out sensitivity analysis for
'GCST90274800.csv' on 'outcome'
